# Differentiation using minimally-invasive bioimpedance measurements of healthy and pathological lung tissue through bronchoscopy

**DOI:** 10.3389/fmed.2023.1108237

**Published:** 2023-04-11

**Authors:** Georgina Company-Se, Lexa Nescolarde, Virginia Pajares, Alfons Torrego, Pere J. Riu, Javier Rosell, Ramon Bragós

**Affiliations:** ^1^Department of Electronic Engineering, Universitat Politècnica de Catalunya, Barcelona, Catalonia, Spain; ^2^Department of Respiratory Medicine, Hospital de la Santa Creu i Sant Pau, Barcelona, Catalonia, Spain

**Keywords:** lung tissue differentiation, minimally-invasive bioimpedance, bronchoscopy, biopsy, respiratory disease

## Abstract

**Purpose:**

To use minimally-invasive transcatheter electrical impedance spectroscopy measurements for tissue differentiation among healthy lung tissue and pathologic lung tissue from patients with different respiratory diseases (neoplasm, fibrosis, pneumonia and emphysema) to complement the diagnosis at real time during bronchoscopic procedures.

**Methods:**

Multi-frequency bioimpedance measurements were performed in 102 patients. The two most discriminative frequencies for impedance modulus (|Z|), phase angle (PA), resistance (R) and reactance (Xc) were selected based on the maximum mean pair-wise Euclidean distances between paired groups. One-way ANOVA for parametric variables and Kruskal–Wallis for non-parametric data tests have been performed with *post-hoc* tests. Discriminant analysis has also been performed to find a linear combination of features to separate among tissue groups.

**Results:**

We found statistically significant differences for all the parameters between: neoplasm and pneumonia (*p* < 0.05); neoplasm and healthy lung tissue (*p* < 0.001); neoplasm and emphysema (p < 0.001); fibrosis and healthy lung tissue (*p* ≤ 0.001) and pneumonia and healthy lung tissue (*p* < 0.01). For fibrosis and emphysema (*p* < 0.05) only in |Z|, R and Xc; and between pneumonia and emphysema (*p* < 0.05) only in |Z| and R. No statistically significant differences (*p* > 0.05) are found between neoplasm and fibrosis; fibrosis and pneumonia; and between healthy lung tissue and emphysema.

**Conclusion:**

The application of minimally-invasive electrical impedance spectroscopy measurements in lung tissue have proven to be useful for tissue differentiation between those pathologies that leads increased tissue and inflammatory cells and those ones that contain more air and destruction of alveolar septa, which could help clinicians to improve diagnosis.

## 1. Introduction

Adequate lung sampling is essential to obtain the diagnosis of lung diseases. Respiratory pathologies can affect the lung parenchyma in a diffuse or localized way. The indication of the most appropriate diagnostic method varies depending on the diagnostic possibilities and the distribution of the pathology. Therefore, the correct characterization of the lung tissue is essential in order to guide the collection of lung samples. Although different imaging methods are currently available (chest CT, PET CT or virtual bronchoscopy), these methods are performed prior to the procedure and do not allow real-time guidance for sample collection. For this reason, advanced bronchoscopic techniques have been developed for a few years, such as the use of radial probe endobronchial ultrasound (radial EBUS) or electromagnetic navigation bronchoscopy (ENB), which allow samples to be obtained from the affected areas in real time (1, 2). However, these are high-cost techniques that are not widely available in Interventional Pulmonology units. The developed line of research aims to expand the diagnostic tools currently available in bronchoscopy with the application of an innovative technique based on the use of bioimpedance data to differentiate between tissue states.

The term bioimpedance (Z) is defined as the opposition offered by a biological tissue to an electrical flow. The bioimpedance is composed by two terms: the resistance (R), which describes the opposition to the electrical flow, mainly in the extracellular and intracellular fluids, of the biological tissue, and the reactance (Xc) which describes the opposition produced by the capacitive behavior of the cell membranes (3, 4). The Xc causes a delay between the voltage and the current causing a phase shift, represented by the phase angle (PA) defined as the tan-1 (Xc/R). Finally, the last parameter that can be derived from the first two is the bioimpedance modulus (|Z|) defined as $\sqrt{R^{2}+Xc^{2}}$ (5). When the bioimpedance data is obtained using a broad band of frequencies it is called electrical impedance spectroscopy (EIS) and it is based on the assumption that at low frequencies the electric current flows through the extracellular medium while at high frequencies the electric current is able to flow through the intra and extracellular medium as it is able to penetrate the cell membranes. Hence, it produces a decrease in |Z|, PA, R and Xc (5).

The use of impedance analysis in studies related to medical field is widely extended, especially for studies of body composition. Previous authors have already applied impedance analysis in lung tissue. Toso et al. (6) compared bioimpedance measurements between healthy lung tissue and neoplasm lung tissue by using an impedance plethysmograph at 50 kHz of frequency obtaining a reduced value of Xc while R value was maintained in neoplasm lung tissue as compared to healthy lung tissue. Baarends et al. (7) studied the accuracy in the prediction of body-water compartments using EIS having the isotope dilution as a reference in patients with severe chronic obstructive pulmonary diseases. Orschulik et al. (8) presented a pilot animal study evaluating the possibility of using EIS to detect acute respiratory distress syndrome. Finally, Meroni et al. (9) used bioimpedance measurements to differentiate among multiple tissues and organs, including lung.

To the extent of author’s knowledge, there are no studies regarding the application of minimally-invasive lung measurements through bronchoscopy to differentiate among different pathologies and healthy lung tissue apart from the previous studies performed by our research group. First, Sanchez et al. (10) described, characterized, calibrated and experimentally validated an EIS instrument for performing minimally-invasive bioimpedance measurements through bronchoscopy. Coll et al. (11) performed tissue differentiation between healthy lung tissue, bronchi and pathological lung tissue obtaining statistical differences among the different groups. However, in that first study, pathologies were not differentiated from each other and all pathological tissues were put into the same group. Riu et al. (12) presented a preliminary artificial intelligence predictive algorithm that was able to discriminate between healthy lung tissue and pathological lung tissue automatically. The three studies carried out to date used the 4-electrode method to acquire the bioimpedance data. That method needed to place 4 electrodes in contact with the lung tissue during all the bioimpedance signal recording.

Occasionally, during bronchoscopy, the patient may have cough or movements that reduce the contact of the impedance catheter with the bronchial wall. To improve contact with the lung surface and decrease measurement time, the authors though that by using the 3-electrode method the bioimpedance signals acquisitions would be more feasible for clinicians. For this reason, the authors performed a study, as presented in Company-Se et al. (13), comparing the capacity to differentiate healthy lung tissue from bronchi using the 4-electrode method and the 3-electrode method. In that study the authors concluded that both methods were able to differentiate between both types of tissue. However, the 3-electrode method had more advantages for the clinical practice, deciding to change the method of signal acquisition. Later, Company-Se et al. (14) presented a method, already used in bioimpedance measures performed in hearth (15), to calibrate the 3-electrode bioimpedance measurements in order to increase tissue differentiation capacity by reducing intra-sample data variability. Moreover, in that study tissue differentiation between healthy lung tissue and neoplasm was performed with significant difference obtained. Finally, they studied bioimpedance differences in healthy lung tissue among smoker, ex-smoker and non-smoker patients, without significance among groups.

The aim of this study is to perform tissue differentiation among healthy lung tissue and pathologic lung tissue from patients with different respiratory diseases (neoplasm, fibrosis, pneumonia and emphysema) through minimally-invasive bioimpedance measurements obtained though bronchoscopy.

## 2. Materials and methods

### 2.1. Participants

Minimally-invasive EIS measures were performed in 102 patients (age: 66 ± 14 years; weight: 74.5 ± 17.2 kg; BMI: 26.8 ± 4.3 kgm^−2^) with a bronchoscopy prescribed between November 2021 and August 2022 at the “Hospital de la Santa Creu i Sant Pau” in Barcelona.

### 2.2. EIS measurements

Electrical impedance spectroscopy measures were taken using the 3-electrode method. A complete description of the measurement system is detailed in Company-Se et al. (14). The lung bioimpedance results from the injection of a multisine current signal (26 frequencies ranging from 1 to 1,000 kHz) between the distal electrode of a tetrapolar catheter (Medtronic 5F RF Marinr) and a skin electrode (3 M Company ref.: 9160F) placed at the level of the ribs. A voltage is induced by the injected current and measured between the distal catheter electrode and a second skin electrode (Ambu BlueSensor VLC ref.: VLC-00-s/10) placed next to the other one. The impedance measures were recorded during a period of 12 s with a sample frequency of 60 spectra per second. Measures were calibrated using a measure of the same patient taken at the bronchi, according to Company-Se et al. (14), to eliminate geometrical factors and reduce intra-group variability.

### 2.3. Measurement protocol

The patients included were evaluated in Interventional Pulmonology Unit. All of them had a complete blood count with coagulation study and radiological evaluation (chest CT or/ and PET CT). A bronchoscopy was indicated to study of respiratory disease. For bronchoscopy, the upper airway was anaesthetized with topical 2% lidocaine; intravenous sedation was provided throughout the procedure with midazolam, fentanil and/or propofol. The acquisition of the bioimpedance data was carried out by inserting the catheter through a port of the bronchoscope. Depending on the respiratory disease, different diagnostic techniques were performed: bronchoaspiration, bronchoalveolar lavage, bronchial brushing, endobronchial or transbronchial biopsy and/or fine needle aspiration. The endoscopic exploration and diagnostic procedures were indicated accordance with the guidelines.

### 2.4. Data analysis

The averaged spectra of the minimally-invasive bioimpedance measured through the 12 s acquisition time was used for tissue differentiation among healthy lung tissue, neoplasm, fibrosis, pneumonia and emphysema. Data was obtained between 1 kHz and 1 MHz. Low frequency values (below 15 kHz) were discarded due to electrode effects and high frequency values (above 307 kHz) were discarded due to capacitive coupling errors. To perform tissue differentiation the frequencies of 15 kHz for |Z| and R and 307 kHz for PA and Xc were chosen based on the calculation of the mean pair-wise Euclidean distance between tissue paired groups. Hence, 15 kHz for |Z| and R and 307 kHz for PA and Xc are the two most discriminative frequencies.

Shapiro–Wilk test was used to assess the distribution of normality of the variables (|Z|, PA, R, Xc and the difference between low and high mean bioimpedance values in |Z|, PA, R and Xc). Normally distributed variables are shown as mean ± standard deviation (SD) and 95% confidence interval (CI) of the mean (lower bound – upper bound). The variables non-normally distributed are shown as median (interquartile range, IQR) and minimum – maximum. One-way analysis of variance (ANOVA, normally-distributed data) with Tamhane *t*2 *post-hoc* test was used to determine statistically significant differences in the |Z|, PA, R and the differences between low and high frequencies mean bioimpedance in |Z|, PA, R and Xc. For non-normally distributed data, Kruskal–Wallis test was used to determine significance in Xc among healthy lung tissue, neoplasm, fibrosis, pneumonia and emphysema.

Discriminant Function Analysis was used to find a linear combination of features that separates healthy lung tissue, neoplasm, fibrosis, pneumonia and emphysema bioimpedance values using the bioimpedance parameters of |Z| and R at 15 kHz and PA and Xc at 307 kHz.

## 3. Results

The total number of bioimpedance samples obtained were 116 (more than one sample was obtained in a few patients) divided according tissue states: 30 healthy lung (age: 62 ± 18 years; weight: 77.7 ± 25.6 kg; BMI: 26.5 ± 4.4 kgm^−2^), 29 neoplasm (age: 69 ± 9 years; weight: 74.3 ± 13.9 kg; BMI: 26.4 ± 4.3 kgm^−2^), 23 emphysema (age: 72 ± 9 years; weight: 72.5 ± 12.3 kg; BMI: 27.3 ± 4.8 kgm^−2^); 12 fibrosis (age: 73 ± 10 years; weight: 76.9 ± 10.7 kg; BMI: 28.4 ± 2.0 kgm^−2^) and 22 pneumonia (age: 62 ± 16 years; weight: 68.9 ± 12.0 kg; BMI: 25.8 ± 4.4 kgm^−2^).

### 3.1. Box plot

**Figure 1:**
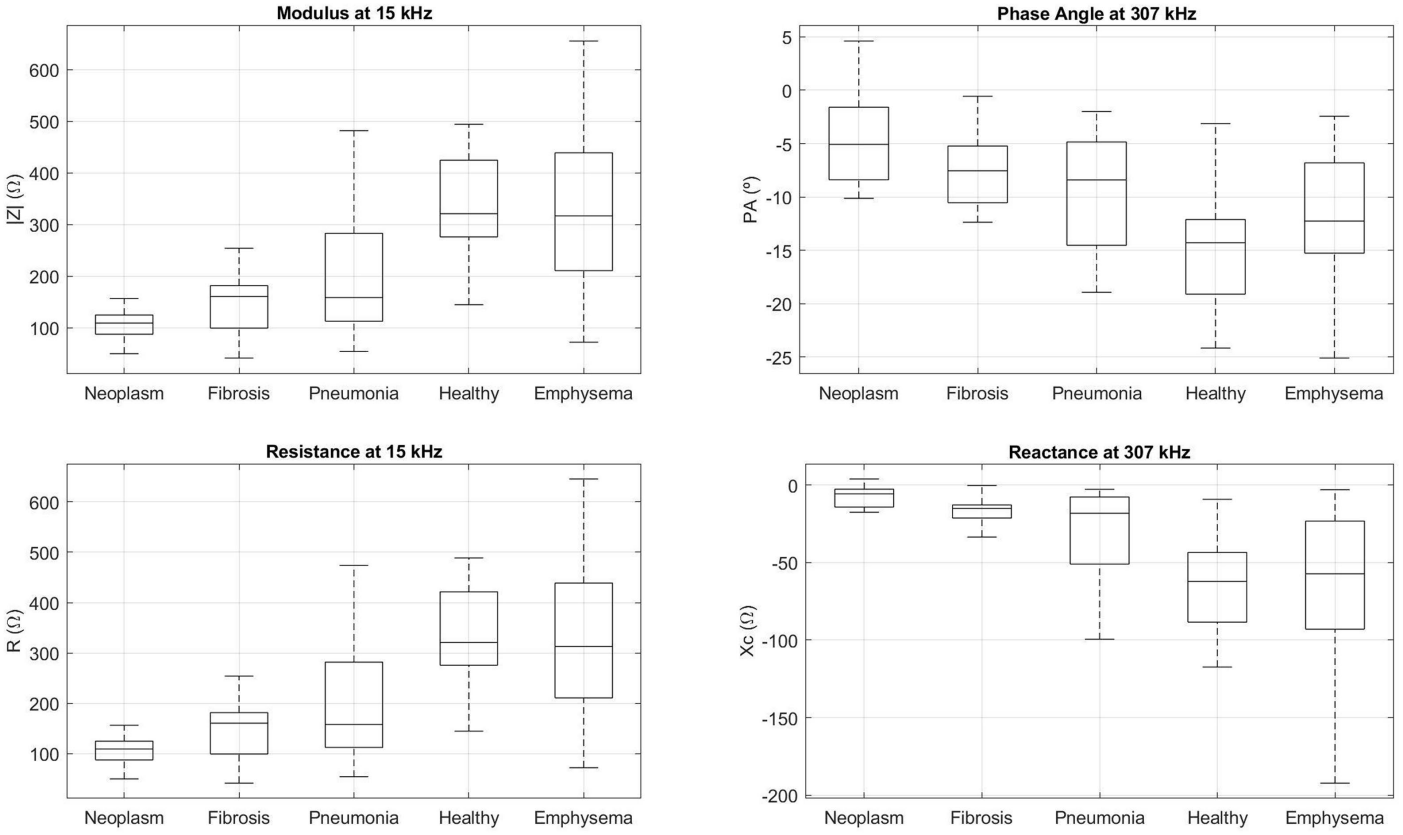
Boxplot of the bioimpedance parameters (|Z| and R at 15 kHz and PA and Xc at 307 kHz) for neoplasm, fibrosis, pneumonia, healthy lung tissue and emphysema tissue samples. The central line of each box represents the median of each group, low and upper box lines represent the 25 and 75 percentiles, respectively, and dashed lines belong to the most extreme points which are not considered outliers.

Figure 1 shows the median (central line of each box) and the 25 and 75 percentiles (down and upper extremes of each box) for |Z| and R at 15 kHz and for PA and Xc at 307 kHz for each of the tissue states [neoplasm (6 small cell lung neoplasm and 23 non-small cell lung neoplasm), fibrosis, pneumonia, healthy lung tissue and emphysema]. Dashed lines represent the most extreme points not considered outliers (1.5 times bigger than the interquartile range). Results show an increase in the |Z| and R and a decrease in the PA and Xc as more air content is the tissue.

### 3.2. Tissue differentiation of minimally-invasive electrical impedance spectroscopy measurements among tissue states

Table 1 lists the descriptive parameters, specified as the mean ± SD, 95% confidence interval for mean (lower bound and upper bound) for normally distributed variables and specified as statistic median (interquartile range, IQR) and minimum – maximum for non-normally distributed variables of |Z|, PA, R and Xc and the results of the one-way ANOVA including the Fisher coefficient (F) and the Kruskal–Wallis test results for the minimally-invasive bioimpedance measures performed in healthy lung tissue (*n* = 30), neoplasm lung tissue (*n* = 29), emphysema (*n* = 23), fibrosis (*n* = 12) and pneumonia (*n* = 22). Both tests show statistical significance (*p* < 0.001) in the |Z|, PA, R and Xc. Fisher coefficient shows higher values in |Z| and R than in PA.

**Table 1:** Descriptions of minimally-invasive bioimpedance measurements for healthy lung tissue, neoplasm, emphysema, fibrosis and pneumonia.

	Healthy (*n* = 30)	Neoplasm (*n* = 29)	Emphysema (*n* = 23)	Fibrosis (*n* = 12)	Pneumonia (*n* = 22)	One way ANOVA test	
						*F*	*p*
|Z| (Ω) 15 kHz	356.29 ± 94.15 (296.47–416.11)	114.53 ± 23.24 (99.77–129.30)	340.54 ± 168.19 (233.68–447.40)	149.17 ± 57.62 (112.56–185.78)	199.12 ± 75.27 (151.29–246.95)	30.80	<0.001
PA (°) 307 kHz	−16.02 ± 4.48 [−18.87 – (−13.18)]	−4.75 ± 4.35 [−7.52 – (−1.99)]	−12.71 ± 6.46 [−16.82 – (−8.61)]	−7.29 ± 3.77 [−9.69 – (−4.89)]	−9.22 ± 4.52 [−12.08 – (−6.35)]	20.52	<0.001
R (Ω) 15 kHz	354.90 ± 93.28 (295.63–414.17)	114.44 ± 23.14 (99.73–129.14)	338.79 ± 166.02 (233.31–444.27)	149.03 ± 57.58 (112.44–185.61)	198.65 ± 75.11 (150.93–246.37)	30.99	<0.001
						Kruskal–Wallis test	
						*p*	
Xc (Ω) 307 kHz	−73.29 ± 29.42 [−92.24 – (−54.86)]	−8.53 ± 7.46 [−13.27 – (−3.79)]	−69.88 ± 52.51 [−103.24 – (−36.51)]	−15.98 ± 9.30 [−21.89 – (−10.07)]	−18.23 (39.35) [−56.63 – (−3.40)]	<0.001	

The variables normally distributed are shown as mean ± SD, 95% confidence interval for mean (lower bound and upper bound) while that non-normally distributed data is shown as statistic median (interquartile range, IQR) and minimum-maximum. In addition, the statistic of the Fisher (*F*) coefficient for variance analysis and the statistical significance (*p*) are also shown.

Table 2 shows the Tamhane *t*2 test results for |Z| at 15 kHz, PA at 307 kHz and for R at 15 kHz. Statistical differences are found between: neoplasm and pneumonia, healthy lung tissue and emphysema; fibrosis and healthy lung tissue and emphysema; pneumonia and healthy lung tissue and emphysema. No statistically significant differences are found between: healthy lung tissue and emphysema; fibrosis and neoplasm; fibrosis and pneumonia.

**Table 2:** Tamhane *t*2 *post-hoc* test results for |Z| at 15 kHz, PA at 307 kHz and R at 15 kHz.

*Post-hoc* Tamhane *t*2 test								
|Z| 15 kHz (Ω)			*R* (Ω) 15 kHz			PA (°) 307 kHz		
		*p*			*p*			*p*
Healthy	Neoplasm	<0.001	Healthy	Neoplasm	<0.001	Healthy	Neoplasm	<0.001
	Emphysema	1		Emphysema	1		Emphysema	0.17
	Fibrosis	<0.001		Fibrosis	<0.001		Fibrosis	<0.001
	Pneumonia	<0.001		Pneumonia	<0.001		Pneumonia	0.008
Neoplasm	Emphysema	<0.001	Neoplasm	Emphysema	<0.001	Neoplasm	Emphysema	<0.001
	Fibrosis	0.247		Fibrosis	0.248		Fibrosis	0.33
	Pneumonia	0.006		Pneumonia	0.006		Pneumonia	0.002
Emphysema	Fibrosis	<0.001	Emphysema	Fibrosis	<0.001	Emphysema	Fibrosis	0.096
	Pneumonia	0.015		Pneumonia	0.014		Pneumonia	0.979
Fibrosis	Pneumonia	0.64	Fibrosis	Pneumonia	0.649	Fibrosis	Pneumonia	0.618

Table 3 shows the pair to pair comparison results for Xc at 307 kHz as Xc is not normally distributed. Statistically significant differences are found between healthy lung tissue and pneumonia, fibrosis and neoplasm, emphysema and fibrosis and neoplasm and between pneumonia and neoplasm.

**Table 3:** Pair to pair comparisons for Xc bioimpedance parameter with significance adjusted by Bonferroni method.

Pair–Pair Comparison Xc (Ω) 307 kHz				
		Statistic	*p*	Adjusted *p*
Healthy	Emphysema	−8.31	0.373	1
	Pneumonia	−33.47	<0.001	0.004
	Fibrosis	−45.28	<0.001	0.001
	Neoplasm	−62.08	<0.001	0
Emphysema	Pneumonia	−25.16	0.012	0.121
	Fibrosis	−36.98	0.002	0.02
	Neoplasm	53.77	<0.001	0
Pneumonia	Fibrosis	11.81	0.328	1
	Neoplasm	28.61	0.003	0.026
Fibrosis	Neoplasm	16.80	0.146	1

### 3.3. Discriminant analysis of minimally-invasive electrical impedance spectroscopy measurements among tissue states

Table 4 shows the Fisher’s linear discriminant functions for lung neoplasm, fibrosis, pneumonia, healthy lung tissue and emphysema bioimpedance measurements and the canonical discriminant functions, by Discriminant Function Analysis of the bioimpedance parameters (|Z|, PA and Xc).

**Table 4:** Fisher’s linear discriminant functions for neoplasm, fibrosis, pneumonia, healthy and emphysema lung bioimpedance samples and canonical discriminant functions for the bioimpedance parameters (|Z|, PA and Xc).

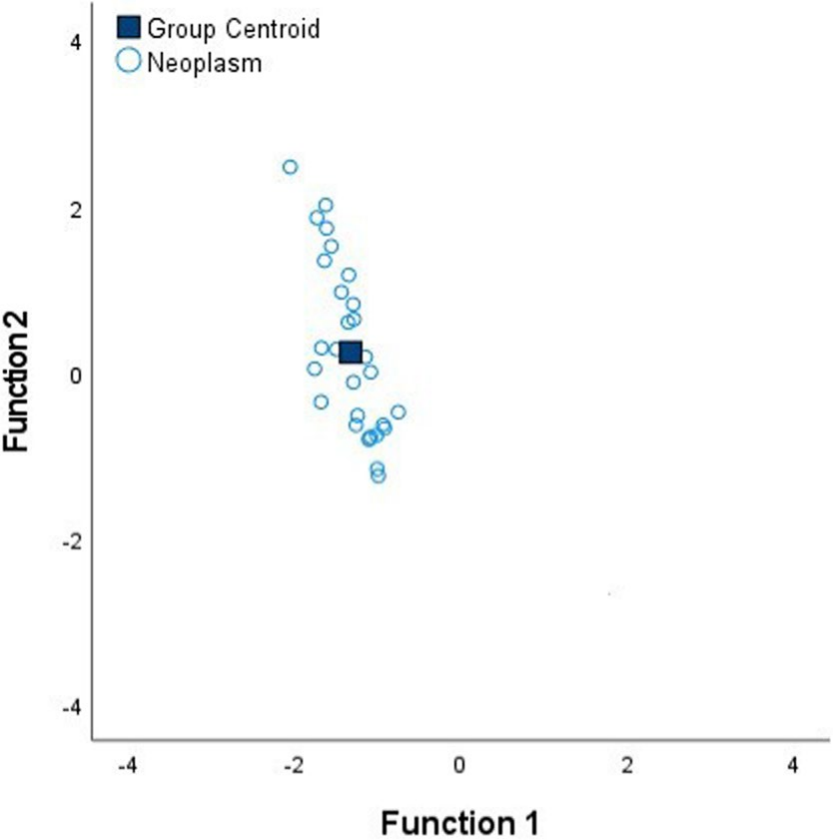	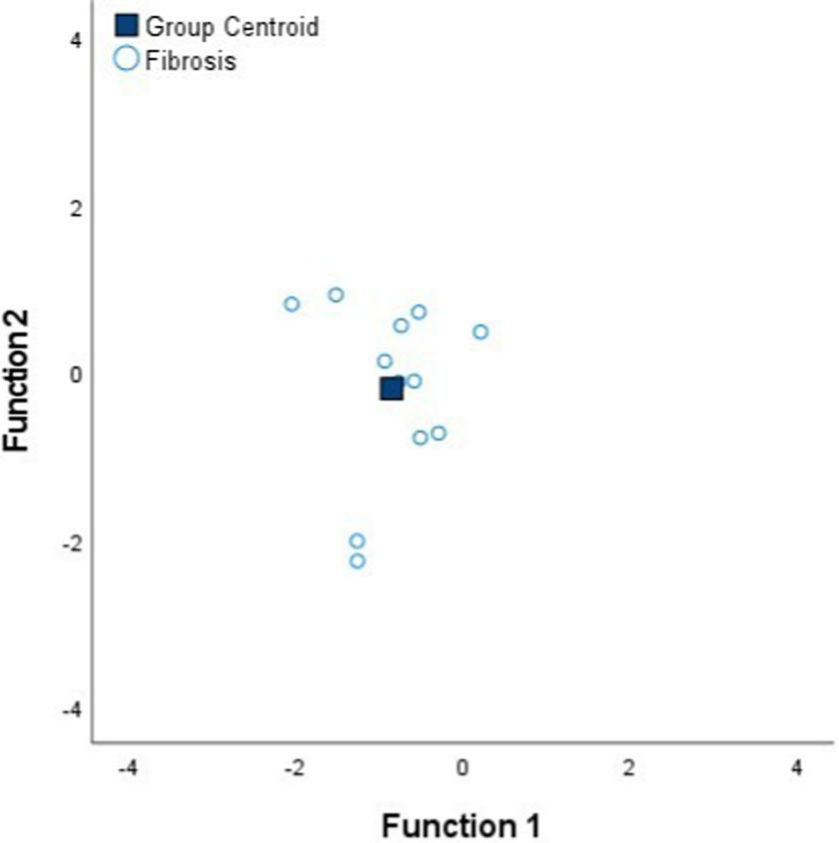	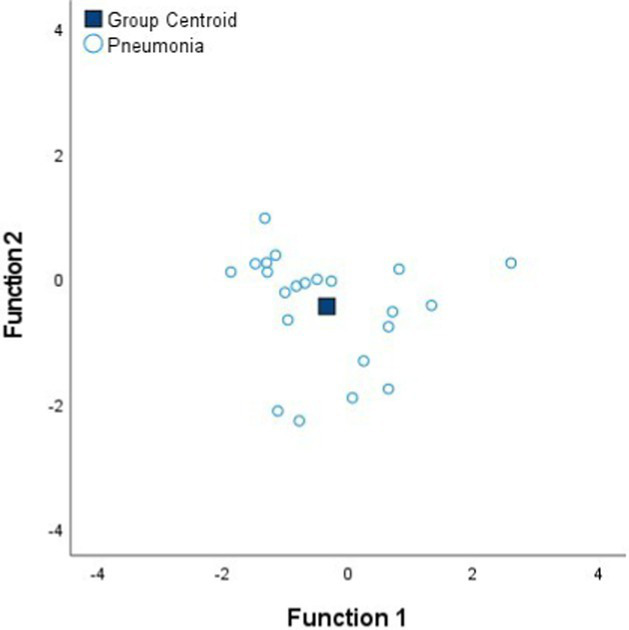
**Fisher’s linear discriminant function**		
$F_{\text{Neoplasm}}=0.03\%\left|Z\right|-0.15\%PA+0.10\%Xc-3.22$	$F_{\text{Fibrosis}}=0.04\%\left|Z\right|-0.29\%PA+0.12\%Xc-4.36$	$F_{\text{Pneumonia}}=0.04\%\left|Z\right|-0.39\%PA+0.12\%Xc-5.38$
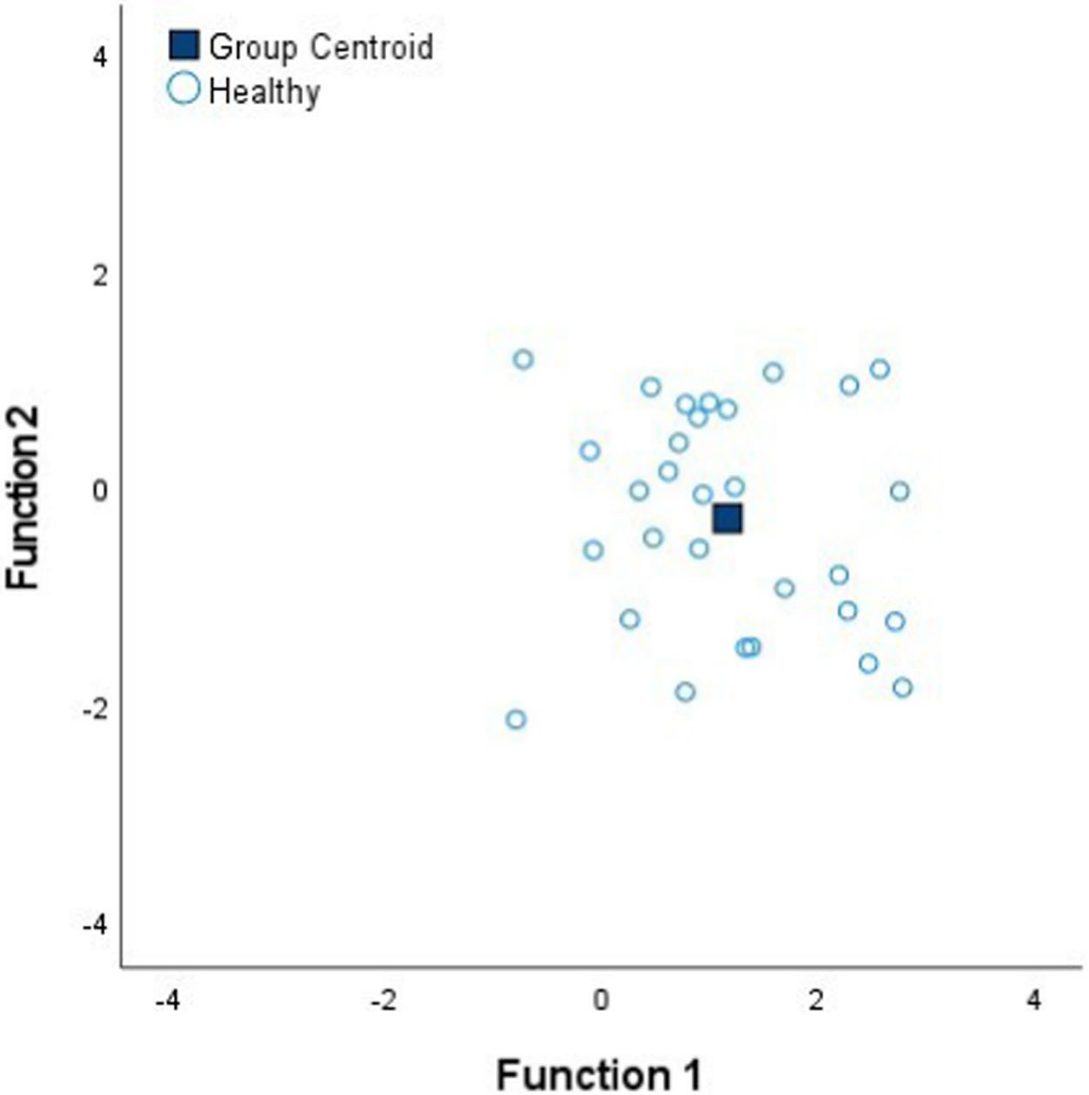	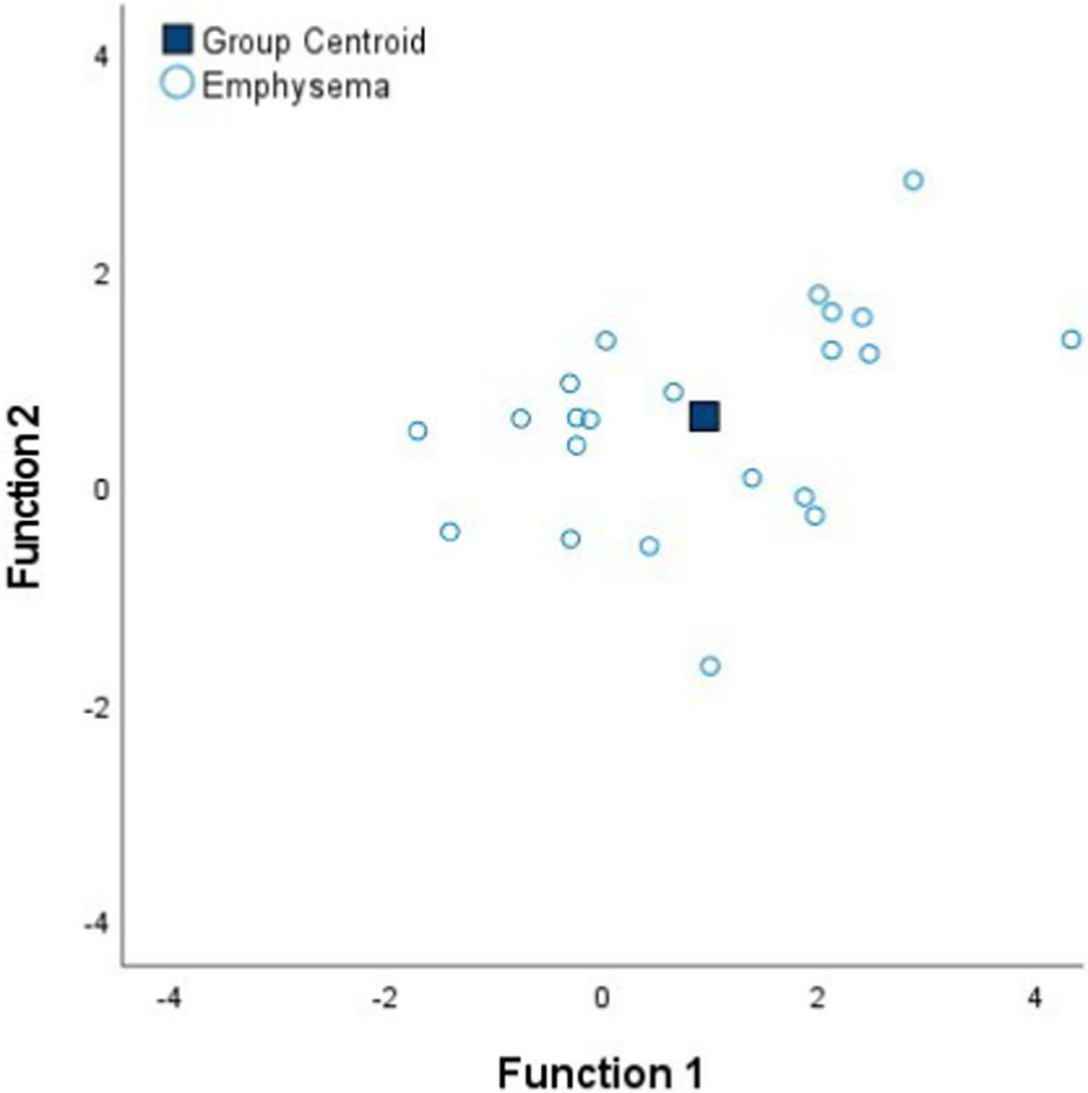	
**Fisher’s linear discriminant function**		
$F_{\text{Healthy}}=0.05\%\left|Z\right|-0.36\%PA+0.12\%Xc-9.59$	$F_{\text{Emphysema}}=0.06\%\left|Z\right|-0.06\%PA+0.10\%Xc-8.37$	
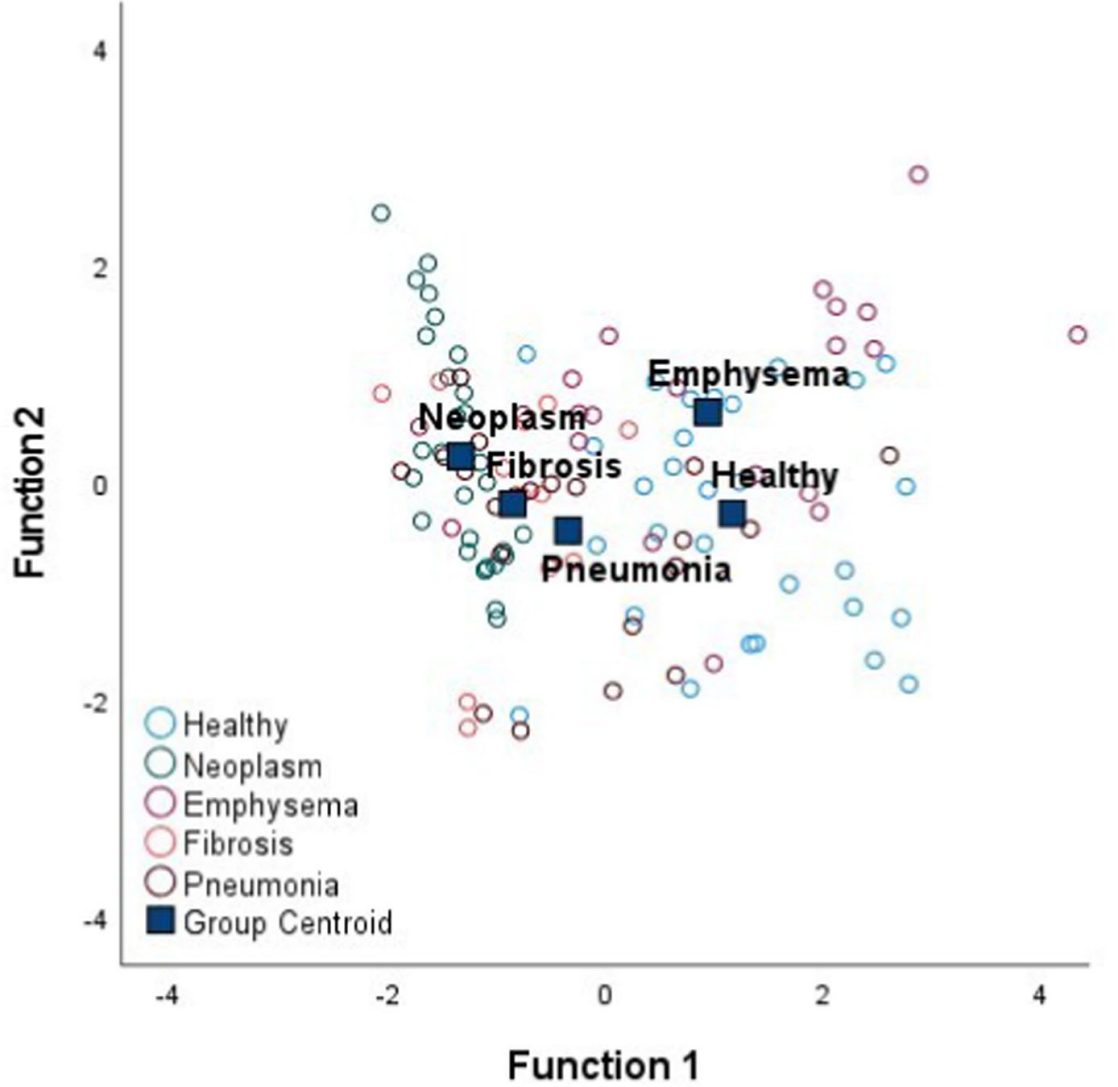		
**Canonical Discriminant Functions**		
FScore1=0.97%|Z|−0.09%PA+0.05%Xc FScore2=0.77%|Z|−1.54%PA+0.51%Xc		

## 4. Discussion

This project developed by the Electronic and Biomedical Instrumentation research group from the Technical University of Catalonia (UPC) and the Interventional Pulmonology Unit from Hospital de la Santa Creu i Sant Pau of Barcelona aims to differentiate among different lung pathologies (neoplasm, fibrosis, pneumonia and emphysema) and healthy lung tissue through minimally-invasive bioimpedance measurements performed directly in lung tissue.

In general terms, in the healthy subject, the lung parenchyma is a histologically heterogeneous structure formed by a network of bronchi and bronchioles that present different subdivisions until generating the alveolar ducts and alveoli where gas exchange occurs. All these structures are surrounded by connective tissue with a reticular structure of collagen and elastic fibers, in which the lymphatic vessels and the capillary of the pulmonary circulation are distributed. In pulmonary pathologies there is an alteration of the pulmonary architecture. The structural changes in the tissues provide a differentiated electrical behavior that will vary according to the tissue involvement and, therefore, could allow differentiating respiratory diseases.

The bioimpedance parameters that can be directly obtained from the bioimpedance measures obtained are the R and the Xc. The first one denotes the behavior of the cellular medium while the second one related to the capacitive behavior of the cell membranes. Two more parameters that can be extracted from the first ones are the |Z| and the PA. While |Z| is highly correlated with the R, the PA is related to Xc (5). To perform tissue differentiation, in this study we have taken into account the four parameters mentioned and have selected the frequency of 15 kHz for |Z| and R and the frequency of 307 kHz for PA and Xc for being the two most discriminative frequencies according to the pair-wise Euclidean distances calculated between pairs of tissue samples. In COPD and specifically in emphysema, the increase in inflammatory cells and oxidative stress produce the secretion of proteases with the capacity to degrade components of the extracellular matrix, which produces direct damage to structural cells and promotes proteolytic degradation of tissues with destruction of alveolar walls (16, 17). The air content present in lungs in proportion to the tissue is higher in this pathology than in the others studied (neoplasm, fibrosis and pneumonia) and also is higher than in healthy lung tissue. In emphysema the data present higher variability and dispersion than in the other tissues studied. This is due to the fact that the sensibility of measure is 2 mm and depending the grade of emphysema the |Z| and R is higher (more air content) or lower. This fact leads to the no differentiation between healthy and emphysema lung tissue.

Considering the above-mentioned, results in Figure 1 show higher values of |Z| and R and lower values of PA and Xc at the tissue samples which have more air content (healthy lung tissue and emphysema). The increase in |Z| and R is due to the non-conductive character of air, as compared with the other tissue samples (neoplasm, fibrosis and pneumonia) in which the proportion of tissue is higher compared to the quantity of air. Related to that condition, the Xc and in consequence the PA, denotes the additional resistance produced by the cell membranes.

The increase of the Xc values in those pathologies in which there is an increase of tissue denotes the increment of cells present in these tissues (neoplasm and fibrosis). In this way, in neoplasm is produced a proliferation of tumor cells and infiltration of lymphatic and blood vessels. The tumor cells have different histological characteristics and, in general, according to the morphology of the cells, lung cancer is classified in non-small cell lung carcinomas (NSCLCs): adenocarcinoma (ADC) and squamous cell carcinoma (SQCC) and small cell lung cancer (18). On the other hand, idiopathic pulmonary fibrosis belongs to the group of diffuse pulmonary diseases (ILD) that includes a heterogeneous classification of pathologies characterized by thickening of the alveolar septa, proliferation of fibroblasts, collagen deposition and, in advanced phases, of the disease, pulmonary fibrosis (19).

For tissue differentiation among neoplasm, fibrosis, pneumonia, healthy lung tissue and emphysema one-way ANOVA test for parametric parameters (|Z|, PA and R) and Kruskal–Wallis test for the non-parametric Xc have been performed. One-way ANOVA test determines differences among different groups means using their variances to determine if their means are equally distributed or not. Hence, a *p* > 0.05 concludes that the distributions of the different groups are equal based on their means. To determine which of the parameters (|Z|, PA or R) have more significance, Fisher coefficient (F) is used, which is defined as the ratio between the variance between samples and the variance within samples. Then, as larger the F, as higher the significance in that variable (20). Also, Kruskal–Wallis test determines differences among groups based on mean ranks (21). Then, according to the results obtained in Table 1, there are statistically significant differences (*p* < 0.001) among tissue samples in |Z|, PA, R and Xc. The significance obtained is higher in |Z| and R than in PA according to the value of the Fisher coefficient obtained. Therefore, a significance higher in |Z| and R means that the most important element for tissue differentiation among the tissue states studied seems to be the proportion of air present in the extracellular medium as compared to the amount of tissue.

Regarding the *post-hoc* tests results (Tables 2, 3), results show statistical significant differences between neoplasm and pneumonia (*p* < 0.05) in |Z|, PA, R and Xc; neoplasm and healthy lung tissue (*p* < 0.001) in |Z|, PA, R and Xc; neoplasm and emphysema (*p* < 0.001) in |Z|, PA, R and Xc; fibrosis and healthy lung tissue (*p* ≤ 0.001) in |Z|, PA, R and Xc; fibrosis and emphysema (*p* < 0.05) in |Z|, R and Xc; pneumonia and healthy lung tissue (*p* < 0.01) in |Z|, PA, R and Xc; and between pneumonia and emphysema (*p* < 0.05) in |Z| and R. No statistically significant differences (*p* > 0.05) are found between neoplasm and fibrosis; fibrosis and pneumonia; and between healthy lung tissue and emphysema.

In summary, as we have described above, neoplasm is characterized by a cell growth and an increase of vascularization and fibrosis is characterized by an increase of tissue in the pathological region despite not being over-vascularized. This similitude of increment of tissue in the pathological region and, in consequence, a decrease in air proportion, lowers the bioimpedance |Z| and R and increases the Xc and PA (Figure 1; Table 1). Despite the over-vascularization of the neoplasm tissue, which lowers slightly the |Z| and R and increases slightly the Xc and PA, the similitude in both pathologies regarding the increment of tissue and, in turn, cell concentration makes not possible to distinguish through minimally-invasive bioimpedance measures between both pathologies. In pneumonia, the inflammatory response is initially characterized by a congestive phase with vascular hyperemia followed by an exudative phase in which the presence of neutrophils and fibrin increases, which can completely occupy the alveolar spaces (22) which decreases the impedance |Z| and R as compared to healthy lung tissue as the quantity of air decreases. However, pneumonia presents higher proportion of air than fibrosis which makes the |Z| and R higher in pneumonia than in fibrosis. Despite the differences between these two pathologies, the characteristic of lung condensation hinders the differentiation between both pathologies.

In complement to the statistical tests performed (one-way ANOVA and Kruskal–Wallis) and following with the tissue differentiation, we have performed a discriminant analysis among the different tissue states (neoplasm, fibrosis, pneumonia, healthy lung tissue and emphysema). Each tissue type obtains a Fisher’s linear discriminant function that aims to find a linear function that maximizes the distance between classes of the projected data means and minimizes the projected within-class variance. The generalization of Fisher’s discriminant function is the canonical discriminant function which have maximum discriminant power to classify among multiple groups. The canonical discriminant functions are vectors of canonical variables composed by linear combinations of the original variables (23). According to the results obtained in Table 4, the graphics of the individual tissue type distributions show an increase in the data dispersion as higher is the air proportion in lungs. Moreover, the results of the graphic where all the tissue types are represented show a higher separation between neoplasm and emphysema and neoplasm and healthy lung tissue (as in Figure 1). Moreover, discriminant analysis shows little distance between neoplasm and fibrosis and between fibrosis and pneumonia, which is in accordance to the results obtained in the statistical tests (Tables 2, 3). Moreover, canonical discriminant functions show that in the first function the |Z| have more importance than the PA and the Xc while in the second function the variable which has more importance is the PA.

The differentiation among the different tissue states using the mean impedance values obtained from the 12 s duration of acquisition signals at 15 kHz for |Z| and R and at 307 kHz for PA and Xc have been proved to be useful for the differentiation between neoplasm and pneumonia, neoplasm and healthy lung tissue, neoplasm and emphysema, fibrosis and healthy lung tissue, fibrosis and emphysema, pneumonia and healthy lung tissue and pneumonia and emphysema. However, it has not been proved to be useful to differentiate between neoplasm and fibrosis, fibrosis and pneumonia and healthy lung tissue and emphysema. As discussed in Company-Se et al. (13) the 3-electrode method was more suitable for the minimally-invasive lung tissue measurements than the 4-electrode method. Furthermore, as also stated in Company-Se et al. (13) the two-electrode method (the simplest method) that only uses the tip of the catheter and an external electrode was discarded because this last method has higher interpatient variability due to non-related lung tissue characteristics factors. Moreover, this last method depends on sweat regulation and skin hydration. Despite the reduction of the data variability through calibration, the data variability continues to be high. This is due to the different respiratory patterns observed in the different patients. While some patients remain still during the signal acquisition, others produce apneas, cough or even movement, affecting the mean values of the signals. In future studies, bronchoscopy will be performed in patients which would undergo general anesthesia in order to study if it affects to the reduction of data variability.

### 4.1. Contribution

The minimally-invasive bioimpedance measurements is a complementary method to bronchoscopy procedure for real time diagnosis through tissue differentiation for respiratory diseases. The major contribution is the capability to differentiate between those pathologies that leads increased tissue and inflammatory cells and those ones that contain more air and destruction of alveolar septa.

### 4.2. Limitation

To validate minimally-invasive bioimpedance as a method for real time diagnosis through tissue differentiation for respiratory diseases, it is necessary to perform studies that compare standardized procedures that allow real-time localization of pulmonary lesions, such as electromagnetic navigation bronchoscopy (ENB) or radial EBUS.

## 5. Conclusion

The use of minimally-invasive bioimpedance measurements have been proven to be useful for tissue differentiation among lung pathologies and healthy lung tissue. Statistical differences have been found between groups by using the two most discriminative frequencies. Bioimpedance has proven to differentiate between those pathologies that leads increased tissue and inflammatory cells and those ones that contain more air and destruction of alveolar septa.

## Data availability statement

The raw data supporting the conclusions of this article will be made available by the authors, without undue reservation.

## Ethics statement

The studies involving human participants were reviewed and approved by “Hospital de la Santa Creu i Sant Pau” (CEIC-73/2020). The patients/participants provided their written informed consent to participate in this study.

## Author contributions

RB, VP, PR, JR, GC-S, AT, and LN designed the experiments and revised the paper and approved the final version of the manuscript. GC-S, VP, and AT performed the experiments. LN and GC-S performed the data processing, analyzed the data, drafted the manuscript and prepared the tables and figures. All authors contributed to the article and approved the submitted version.

## Funding

This work was supported by the Spanish Ministry of Science and Innovation (PID2021-128602OB-C21) and supported by the Secretariat of Universities and Research of the Generalitat de Catalunya and the European Social Fund.

## Conflict of interest

The authors declare that the research was conducted in the absence of any commercial or financial relationships that could be construed as a potential conflict of interest.

## Publisher’s note

All claims expressed in this article are solely those of the authors and do not necessarily represent those of their affiliated organizations, or those of the publisher, the editors and the reviewers. Any product that may be evaluated in this article, or claim that may be made by its manufacturer, is not guaranteed or endorsed by the publisher.
